# Parvalbumin Interneuron Activation-Dependent Adult Hippocampal Neurogenesis Is Required for Treadmill Running to Reverse Schizophrenia-Like Phenotypes

**DOI:** 10.3389/fcell.2020.00024

**Published:** 2020-02-04

**Authors:** Yandong Yi, Yuanlong Song, Yisheng Lu

**Affiliations:** ^1^Department of Physiology, School of Basic Medicine, Huazhong University of Science and Technology, Wuhan, China; ^2^Department of Pharmacy, Wuhan No. 1 Hospital, Tongji Medical College, Huazhong University of Science and Technology, Wuhan, China; ^3^Institute of Brain Research, Collaborative Innovation Center for Brain Science, Huazhong University of Science and Technology, Wuhan, China

**Keywords:** treadmill running, adult neurogenesis, hippocampus, schizophrenia, parvalbumin, ErbB4

## Abstract

Physical exercise can alleviate some of the schizophrenia symptoms in patients, the mechanisms, however, are still unclear. To investigate whether the GABAergic interneuron involved in the therapeutic effect of treadmill running on schizophrenia, the parvalbumin (PV)-positive GABAergic interneurons in the dentate gyrus (DG) was specifically activated or abolished and the effects were evaluated. In the MK801-induced schizophrenia-like animal model, we found:(1) Treadmill running rescued the schizophrenia-related behavioral phenotypes, promoted the adult hippocampal neurogenesis, and increased the dendrite number and complexity of newborn neurons. (2) Treadmill running increased the number of PV-positive interneurons in the DG; genetic ablation of these interneurons reduced adult neurogenesis and abolished the effect of treadmill running on the schizophrenia-related behaviors. Consistently, chemogenetic activation of these interneurons improved neurogenesis and alleviated the schizophrenia-related behaviors. These results suggest a pivotal role of PV-positive interneuron-mediated adult neurogenesis in exercise. (3) However, schizophrenia-related behavioral phenotypes and adult neurogenesis in the DG could still be reversed by exercise after specifically knocking out the schizophrenia-related gene ErbB4 in PV interneurons, as a means to reduce their GABA release. These results suggest that activation of PV interneurons in the DG is sufficient for treadmill running to reverse schizophrenia-like phenotypes.

## Introduction

Schizophrenia is a serious chronic mental disorder that affects approximately 1% of the world’s population since their adolescence or early adulthood, thus placing a high economic burden for individuals and society (Wu et al., 2006; Chang et al., 2017). Schizophrenia symptoms can be classified into three categories: positive, negative, and cognitive. Positive symptoms like hallucinations and delusions are treatable with antipsychotic drugs, whereas negative symptoms like affection blunt and cognitive symptoms are almost not amenable with drugs (Goldberg et al., 2007). Clinical investigations have indicated that physical exercise is effective in alleviating negative and cognitive symptoms in patients with schizophrenia (Rosenbaum et al., 2014; Firth et al., 2015; Dauwan et al., 2016), but the mechanism is largely unknown.

Adult neurogenesis is crucial for normal cognitive function and regarded as a unique brain plasticity that occurs mainly in two regions, the subgranular zone (SGZ) of the hippocampal dentate gyrus (DG) and the subventricular zone (SVZ). Studies from postmortem (Pedersen and Cohen, 1990; Falkai et al., 2016), fibroblasts-derived induced pluripotent stem cell (iPSC) from patients with schizophrenia (Yu et al., 2014), and genetic or pharmacological animal models of schizophrenia (Pereira et al., 2007; Christian et al., 2014; Allen et al., 2016) have revealed that dysregulation of adult neurogenesis in the SGZ of the DG is associated with schizophrenia; adult hippocampal neurogenesis impairment can induce schizophrenia-related behavioral phenotypes, especially cognitive symptoms (Reif et al., 2006; Mao et al., 2009), and promotion of adult neurogenesis in the SGZ can rescue behavioral deficits in schizophrenia animal models (Ouchi et al., 2013; Wu et al., 2017).

Although both typical and atypical antipsychotics used for schizophrenia cannot promote adult neurogenesis in animal models (Toro and Deakin, 2007) and patients (Reif et al., 2006), physical exercise promotes adult neurogenesis in the SGZ and SVZ (Pajonk et al., 2010); however, whether exercise improves schizophrenia-related phenotypes through promotion of adult neurogenesis needs further investigation. Several neurotransmitters and neurotrophic factors are involved in the effect of exercise in promoting adult neurogenesis. Among them, GABA has attracted much attention in the context of the transition from excitatory to inhibitory during maturation of neurons (Yamashita and Fukuda, 1993; Moss and Toni, 2013), which is regulated by Disrupted-in-Schizophrenia 1 (DISC1) (Kim et al., 2012) and brain-derived neurotrophic factor (BDNF) signaling (Rivera et al., 2002). However, how local GABAergic interneurons regulate neural stem cells (NSCs) is still not well understood; probably due to the distinct properties of different types of GABAergic interneurons.

In the DG, parvalbumin (PV)-, somatostatin-, and vasoactive intestinal polypeptide-containing neurons are the main types of GABAergic interneurons, with PV interneurons being particularly critical due to their fast firing pattern and projection to the soma or the axon initial segments of target neurons. Schizophrenia is associated with abnormalities in PV interneurons, resulting from their weakened inhibitory control of mature pyramidal cells (Beasley et al., 2002; Zhang and Reynolds, 2002; Sakai et al., 2008; Lewis et al., 2012). PV interneurons can also form immature synaptic inputs onto the already-born progeny, thus promoting its survival and development in the adult DG (Song et al., 2013); however, PV neurons inhibit quiescent NSC activation (Song et al., 2012). Thus, it is still largely unknown whether PV-positive interneurons’ activity can alleviate schizophrenia-like phenotypes through modulation of adult neurogenesis.

To date, more than one hundred schizophrenia-related genes have been identified, many of them are expressed in PV neurons with hypofunction and, as a result, lead to reduced GABA release from these neurons. Neural trophic factor neuregulin 1 (NRG1) and its receptor ErbB4 are two schizophrenia-related genes (Stefansson et al., 2002; Yang et al., 2003; Mei and Xiong, 2008). ErbB4 is specifically expressed in GABAergic neurons in rodents and mainly expressed in PV neurons (Fazzari et al., 2010). Specific knockout of ErbB4 in PV neurons reduces GABA release (Lipska, 2004; Neddens and Buonanno, 2010). If PV interneurons in the DG are required for the effect of exercise therapy in schizophrenia, whether ErbB4 in PV interneurons in the DG is also required for the effect of exercise therapy to alleviate schizophrenia-like phenotypes through modulation of adult neurogenesis needs further investigation.

MK801 is an antagonist of *N*-methyl-D-aspartate (NMDA) receptor often used to induce schizophrenia-like phenotypes in animal models, although the mechanisms are still largely unknown (Uttl et al., 2018). MK801 injection has been shown to significantly decrease the number of PV-positive neurons (Braun et al., 2007; Kaneta et al., 2017) and the expression of PV mRNA in the DG (Romon et al., 2011). Chronic MK801 treatment decreases adult neurogenesis (Maeda et al., 2007), whereas acute treatment enhances neurogenesis in the DG (Cameron et al., 1995; Singh et al., 2017), suggesting that chronic treatment is a good means to investigate adult neurogenesis in schizophrenia.

In this study, we found in MK801-induced schizophrenia-like animal models, adult neurogenesis and schizophrenia-related behavior phenotypes could be rescued by treadmill running. To investigate whether PV interneurons is required for therapy effect of treadmill running, PV interneurons were ablated or activated through induced apoptosis or chemogenetic method, respectively. Ablating PV interneurons in the DG decreased adult neurogenesis and blocked the rescue effect of treadmill running, while activation of these neurons alleviated schizophrenia-related phenotypes and improved neurogenesis. These data suggest that PV interneurons are required for the effects of treadmill running in increasing adult neurogenesis and rescuing schizophrenia-like phenotypes. However, treadmill running could still rescue schizophrenia-like phenotypes after knockout of ErbB4 in PV neurons, suggesting that ErbB4 in PV neurons in the DG is not required for the effect of exercise therapy in schizophrenia.

## Materials and Methods

### Animals

All experimental procedures with mice were approved by the Animal Welfare Committee of Huazhong University of Science and Technology, and experiments in this study were carried out in accordance with the rules of this committee.

The mouse strains used included wild-type C57BL/6, PV-Cre, and loxP-flanked (floxed)-ErbB4 mice. C57BL/6 male mice (7-weeks-old) were purchased from the Experimental Animals Center of Tongji Medical College, Huazhong University of Science and Technology. Floxed-ErbB4 (Garcia-Rivello et al., 2005) and PV-Cre mice were described previously (Wen et al., 2010), PV-Cre mouse line used was kindly provided by Dr. Lin Mei (Case Western Reserve University).

Parvalbumin-Cre mice were crossed with floxed-ErbB4 mice to generate PV-Cre;floxed-ErbB4^+/+^ (PV-ErbB4^–/–^) mice, in which ErbB4 was ablated in PV neurons. Tail genomic DNA was used for genotyping by PCR. PV-ErbB4^–/–^ and ErbB4^+/+^ mice were confirmed by PCR. A 500-bp DNA fragment (mutant) was specifically amplified in ErbB4^+/+^ mice, while a 300-bp fragment was detected for the PV-Cre allele.

Less than 5 mice were housed per cage at 22–24°C and 55–80% humidity, on a schedule of 12:12 h light/dark cycle, with water and food available *ad libitum*. All mice were backcrossed with C57BL/6 mice for more than 10 generations.

### Treadmill Running

Mice in the run groups underwent adaptive run-training sessions in individual lanes of a treadmill (FT-200, Taimeng, China; 5 m/min for 45 min) for 5 days, and running sessions (5 m/min for 10 min, 8 m/min for 30 min, 5 m/min for 10 min) for the next 4 weeks to prevent stress-induced inhibition of hippocampal neurogenesis (Leem et al., 2018). Mice in the static groups were left for the same duration on the treadmill without running.

### Stereotaxic Viral Injection

Adult mice were anesthetized with chloral hydrate (400 mg/kg, i.p.) and head-fixed in a stereotaxic device (RWD life science; 68025). Viruses were bilaterally injected (0.2 μL per side, 20 nL/min) with a glass pipette (Cetin et al., 2006) (tip size, ∼20 mm) at the following coordinates relative to bregma: anteroposterior, −1.94 mm; dorsoventral, −2.14 mm; and mediolateral, ± 1.5 mm. After injection, the glass pipette was left in place for 10 min before slowly removing it. The titers of AAV-flex-taCasp3-TEVp (Taitool Bioscience, catalog #S0236-9), shRNA-EF1a(S)-EGFP (Obio Technology, Shanghai, China) and AAV-DIO-hM3DGq-mCherry (GeneChem technologies, catalog #AAV00030) were 10^12^ genome copies per mL. After injection of AAV-DIO-hM3DGq-mCherry into the DG of PV-Cre mice, hM3DGq was expressed only in PV interneurons in the DG (PV-DG^hM3Dq^ mice). Mice were injected with either saline or clozapine-N-oxide (CNO, dissolved in saline, 3 mg/kg), an agonist for the DREADD (Designer Receptors Exclusively Activated by Designer Drug) system, 30 min prior to the behavioral tests.

### Behavioral Analysis

Only 12-week-old males were used. The analysis was carried out by investigators unaware of the animal genotype and grouping information. Tests were performed in a sequential order as follows: open-field test (OFT), prepulse inhibition (PPI), T-maze test, and fear conditioning. All tests were performed during the light period, and all mice were handled for at least 5 min twice a day for 3 days prior to the behavioral test. No body weight, whisker number, and motor coordination differences were found in any groups during behavioral analysis.

### Open Field Test

Open field test (OFT) is a widely used test to determine locomotion, exploratory and anxiety-related behavior in rodent models of CNS disorders. Hyperactivity, a characteristic rodent phenotype corresponding to the psychomotor agitation of schizophrenic patients (Adriani et al., 1998; Carey et al., 1998; Kanes et al., 2007), is commonly tested by OFT. The mice were gently placed at the center of a rectangular chamber (45 × 45 × 45 cm), and movement was monitored for 5 min using an automated video tracking system (TMV-100S, TaiMeng, China). After each trial, the apparatus was swept with 75% alcohol to avoid the presence of olfactory cues. The distance traveled during a session was measured.

### Prepulse Inhibition of Acoustic Startle

Prepulse inhibition (PPI) deficits seen in patients with schizophrenia can be mimicked in rodents by treatment with MK801 (Curzon and Decker, 1998; Swerdlow and Geyer, 1998; Eyjolfsson et al., 2006). PPI tests were conducted in a sound-attenuated box (SR-LAB, Startle Response System, San Diego Instruments, CA). Mice were placed in a non-restrictive Plexiglas cylinder mounted on a plastic platform, and their motion was transduced into analog signals via a piezoelectric accelerometer. Before the test, mice were allowed to habituate to the chamber, to a 70-dB background white noise for 5 min, and to auditory-evoked startle stimuli (120 dB, 20 ms for 10 times). In the PPI test, mice were subjected to 12 startle trials (120 dB, 20 ms) and 12 prepulse/startle trials (20 ms, white noise at 75, 80, or 85 dB at 100-ms intervals; and 20 ms, 120-dB startle stimulus). Different trial types were presented pseudorandomly, with each trial type presented 12 times, while no two consecutive trials were identical. Mouse movement was measured during 100 ms after the startle stimulus onset (sampling frequency 1 kHz) for 100 ms. PPI (%) was calculated according to the formula: [1 - (startle amplitude on prepulse-pulse trials/startle amplitude on pulse alone trials)] × 100%.

### T-Maze Test

Working memory deficits are considered important for poor cognitive performance in schizophrenia (Seidman et al., 1994; Tek et al., 2002). T-maze test is widely used for evaluation of working memory. The apparatus was an enclosed maze with three arms, a start arm (38 × 7 cm) and two symmetrical choice arms (30 × 7 cm) flanking a central choice area (7 × 7 cm). In each session, the mouse was placed in the start arm facing the wall and allowed to explore the apparatus. As soon as the animal entered (with all four paws) one of the two choice arms, the door of that compartment was closed for 30 s. Then the mouse was removed gently from the maze and placed in the home cage, then placed back in the start arm to perform a second choice trial. The novel arm in the second trial was the right choice. The test was performed in the room where the animals were housed and comprised 10 trials. Correct percentage (%) = (total entry to the novel arm in the second trial/total second trial) × 100%.

### Contextual Fear Discrimination Learning

This paradigm tests the animal’s ability to distinguish between two similar contexts (Sahay et al., 2011). Pattern separation is a fundamental computational function of the DG (McNaughton et al., 1986; Faghihi and Moustafa, 2015), which depends on normal adult neurogenesis.

The shock-associated training context A (shock) and the similar (no-shock) context B shared many features, including an exposed stainless steel grid floor and roof. The similar context differed from the training context in that four plastic inserts were used to cover the walls. A non-alcoholic antiseptic solution was used to clean the grids between trials. In pilot experiments, mice were exposed to the training context where they received a single 2-s foot shock of 0.75 mA, 185 s following placement in the sound proof chamber (29 × 29 × 24 cm; Coulbourn instruments, Allentown, PA, United States, model H10-11M-7C-SF). For discrimination learning, mice were again exposed to training context A. One hour later, mice were placed in the similar context and left for 180 s, and were never shocked. Freezing levels were measured by video camera each day and computed as a Discrimination ratio: (Freezing training context - Freezing similar context)/(Freezing training context + Freezing similar context). A score of 0 indicated complete lack of discrimination, i.e., freezing levels were the same in the similar and training contexts (Freezing similar context = Freezing training context).

### Immunofluorescence

Wild-type and PV-Cre mice were anesthetized with chloral hydrate and perfused transcardially with 4% paraformaldehyde (PFA) in phosphate buffered saline (PBS). Brains were fixed overnight in 4% PFA at 4°C. After cryoprotected in 30% sucrose, brains were frozen in OCT medium (Tissue-Tek, Sakura, Japan) and sliced at 40-μm free-floating coronal sections using a cryostat (Thermo Scientific, HM550).

Sections were rinsed three times in PBS (pH 7.4) and blocked in blocking buffer (PBS with 0.1% Triton containing 10% goat serum and 3% BSA) for 60 min at room temperature. Sections were then incubated at 4°C overnight in blocking buffer containing the following primary antibodies: rabbit anti-PV (A2791, Abclonal; 1:100), rabbit anti-Ki67 (ab15580, Abcam; 1:500), rat anti-BrdU (FITC conjugated; ab74545, Abcam; 1:300), mouse anti-NeuN (ab104224, Abcam; 1:500). After washing with PBS three times, sections were incubated with donkey anti-rabbit IgG conjugated with Alexa Fluor 594 or goat anti-rabbit IgG conjugated with Alexa Fluor 488 in blocking buffer for 1 h at room temperature. Samples were mounted with mounting medium (containing DAPI), and images were taken using Olympus Fluoview FV1000. Quantification of labeling was determined by counting all fluorescent cells in every sixth section. A total of five images were analyzed per mouse, and each group contained 5 mice.

For BrdU staining, sections were incubated with 2 N HCl for 30 min at 37°C to denature the DNA, followed by neutralization with 0.1 M borate buffer (pH 8.5) for 10 min at room temperature. After neutralization, sections were rinsed with PBS several times before incubation with primary antibodies.

Quantification of labeling was determined by counting all fluorescent cells in every section. A total of five section were analyzed in each mouse, and each group contained five mice.

### Sholl Analysis

The total dendritic branches in one GFP-positive cell in DG were chosen and scanned by an Olympus Fluoview FV1000. Images were imported into ImageJ by 8 bit and made binary. Dendritic branches was analyzed by the ImageJ Sholl Analysis Plugin^1^. The center of all concentric circles defined as the center of cell soma. There were three mice in each group, five sections in each animal were picked. Three cells in the DG from each section were analyzed and counted.

### Western Blot

One day after the behavioral tests, mice were anesthetized with 5% chloral hydrate (8 mL/kg), and the tissues were rapidly collected. Tissue homogenates were prepared on ice in RIPA buffer containing 50 mM Tris–HCl (pH 7.4), 150 mM NaCl, 5% sodium deoxycholate, 1% NP40, 1 mM PMSF, and 1 μg/mL protease inhibitor cocktail. Homogenates or bound proteins were resolved on SDS/PAGE and transferred to PVDF membranes, which were then incubated in blocking buffer [tris-phosphate buffer solution (TBS) containing 0.1% Tween-20 and 5% milk] for 1 h at room temperature before adding the primary antibodies for incubation overnight at 4°C. After washing, the membranes were incubated with horseradish peroxidase-conjugated secondary antibodies (BL003A, Biosharp; 1: 30000) in TBS for 1 h at room temperature. Immunoreactive bands were visualized using enhanced chemiluminescence (1705060, Biorad). Films were scanned using MicroChemi 4.2 (DNR Bio-imaging Systems, Israel). Primary antibodies used were: rabbit anti-ErbB4 (A10853, Abclonal; 1: 1000), rabbit anti-parvalbumin (A2791, Abclonal; 1: 1000), and rabbit polyclonal anti-a-tubulin (AC003, Abclonal; 1: 500). The band density was measured by ImageJ software [National Institutes of Health (NIH), United States] and Data were analyzed by GraphPad Prism 6.0.

### Statistical Analysis

All statistical analysis was performed using GraphPad Prism 6.0 (GraphPad Software, San Diego, CA). Statistical differences between two groups were analyzed by applying the two-tailed Student’s *t*-test. Data containing more than two groups were tested by using analysis of variance (ANOVA). Significant main effects or interactions were followed up with Tukey’s *post hoc* test. Data are presented as mean ± standard error of the mean. Statistical differences were considered to be significant when *P* < 0.05.

## Results

### Treadmill Running Increases DG Adult Neurogenesis of MK801-Induced Schizophrenia Mice

**FIGURE 1:**
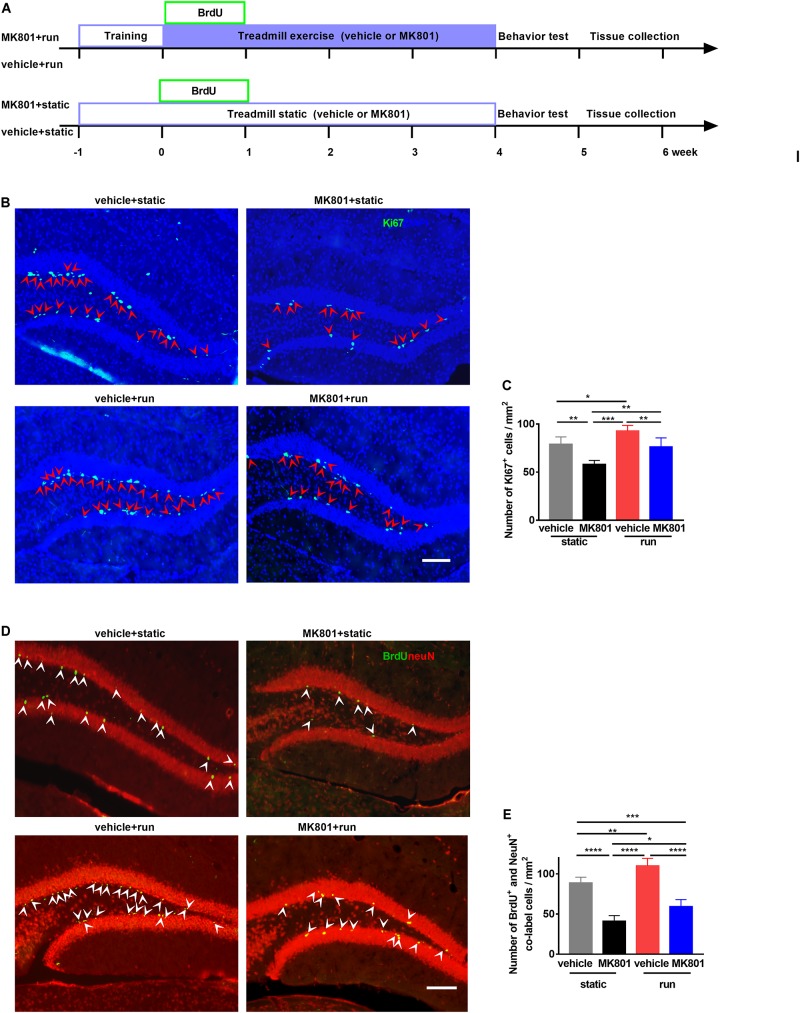
Treadmill running increases adult neurogenesis in the dentate gyrus (DG) in the MK801-induced schizophrenia-like mouse model. **(A)** Schematic experimental design of BrdU injection for the treadmill running in MK801-induced schizophrenia-like model. **(B)** Representative photomicrographs showing Ki67- and (DAPI-positive cells in the DG. Arrowheads (in red) indicate co-labeled cells (Ki67^+^DAPI^+^ cells) in the DG. Scale bar, 100 μm. **(C)** Quantitative analysis of the number of Ki67-positive cells in the DG. Data are expressed as mean ± SEM, and there were five mice in each group. Five sections in each animal were picked and counted. The numbers of Ki67 + cells in the vehicle + static, MK801 + static, vehicle + run and MK801 + run groups were 78.85 ± 7.81, 58.06 ± 4.15, 92.7 ± 45.77 and 76.16 ± 9.46 cells/mm^2^, respectively, One-way ANOVA, *F*_3_,_16_ = 20.21, *P* < 0.001; *post hoc* test: *P < 0.05, **P < 0.01, ***P < 0.001. **(D)** Representative photomicrographs showing BrdU- and NeuN-positive cells in the DG. Arrowheads (in white) indicate co-labeled cells (BrdU^+^NeuN^+^ cells) in the DG. Scale bar, 100 μm. **(E)** Quantitative analysis of the number of BrdU^+^NeuN^+^ cells in the DG. Data are expressed as mean ± SEM, and there were five mice in each group. Five sections in each animal were picked and counted. The numbers of BrdU^+^NeuN^+^ cells in the vehicle + static, MK801 + static, vehicle + run and MK801 + run groups were 88.71 ± 7.27, 40.99 ± 7.17, 109.97 ± 9.38, 59.14 ± 8.88 and 76.16 ± 9.46 cells/mm^2^, respectively. One-way ANOVA, *F*_3_,_16_ = 69.27, *P* < 0.001; *post hoc* test: *P < 0.05; **P < 0.01, ***P < 0.001, ****P < 0.0001.)

To investigate the effect of treadmill running on MK801-induced schizophrenia model, MK801 (HY-15084, MCE, 0.5 mg/kg body weight) or saline were administered intraperitoneally (i.p.) 2 h prior to each treadmill running. Animals were randomly selected and divided into four groups according to the treatment: (1) vehicle + static: mice subjected to saline injection and static treadmill, (2) MK801 + static: mice subjected to MK801 injection and static treadmill, (3) vehicle + run: mice subjected to saline injection and running treadmill, (4) MK801 + run: mice subjected to MK801 injection and running treadmill (Figure 1A). In agreement with previous studies (Overall et al., 2013), our investigation indicated that running promotes neurogenesis in the DG, comparing vehicle + run to vehicle + static group. Ki67-positive cell was used as marker of neurogenesis, these cells were majorly located in the SGZ, in pairs or clusters (Figure 1B). Mice with MK801 (MK801 + static) showed Ki67-positive cells loss in the DG when compared to mice with vehicle (vehicle + static). MK801 administration reduced the proliferation of NSCs in the hippocampus, while treadmill running reversed this effect of MK801 (Figure 1C).

To further investigate whether running affects the survival of NSCs, bromodeoxyuridine (BrdU, Sigma) was injected i.p. to the mice for the first 5 running days in order to label proliferating progenitor cells (Figure 1A). Co-labeling, with anti-NeuN and anti-BrdU antibodies, showed BrdU^+^ cells with an elliptical shape distributed from basal to apical portions of the GCL (Figure 1D). *Post hoc* analysis revealed that MK801 injection significantly decreased NeuN^+^BrdU^+^ cell numbers compared to vehicle injection, while running significantly enhanced NeuN^+^BrdU^+^ cell numbers (Figure 1E). These results indicate that running increases adult neurogenesis in the DG of the schizophrenia-like mouse model.

### Treadmill Running Increases the Number of PV-Positive Interneurons and NSCs in the DG of MK801-Induced Schizophrenia Model Mice

**FIGURE 2:**
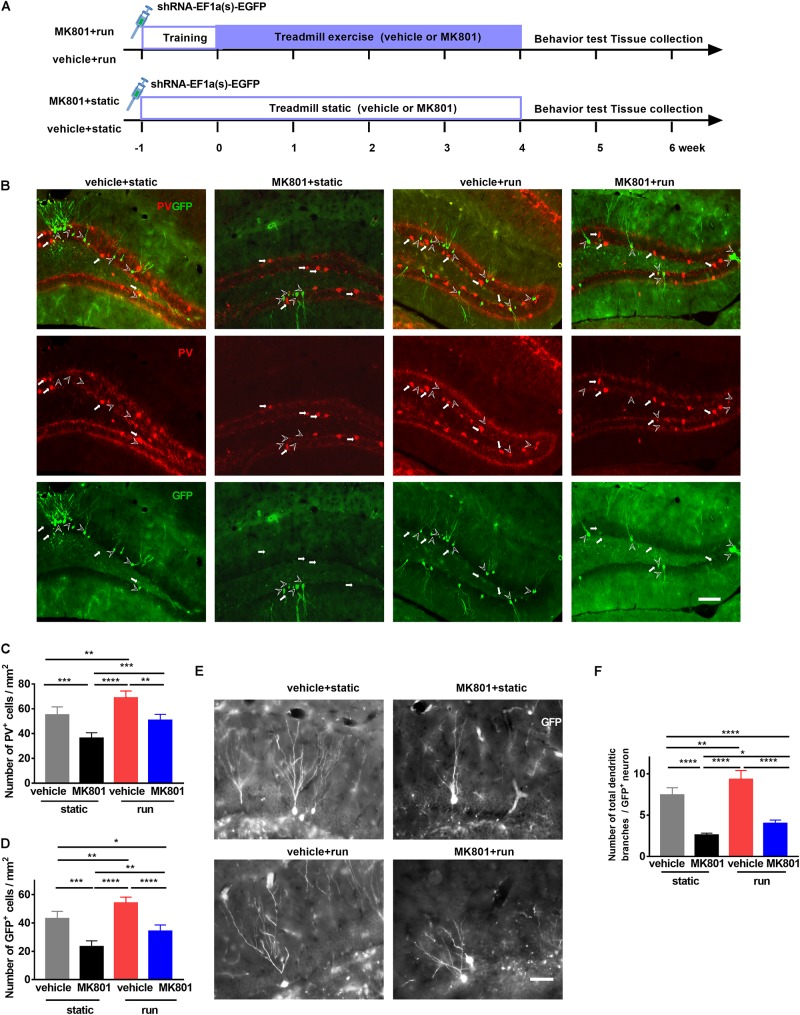
Treadmill running increases newborn cells and parvalbumin-positive (PV-positive) interneurons in the dentate gyrus (DG) of the MK801-induced schizophrenia-like mouse model. **(A)** Schematic experimental design of retrovirus injection for the treadmill running in MK801-induced schizophrenia-like model. **(B)** Representative photomicrographs showing PV-positive interneurons (in red) and GFP-positive cells (in green) in the DG. Arrowheads (in white) indicate (GFP-positive cells. Arrows (in white) indicate PV-positive interneurons. Scale bar, 100 μm. **(C)** Quantitative analysis of PV-positive interneuron number in the DG. Data are expressed as mean ± SEM, and there were five mice in each group. Five sections in each animal were picked and counted. The numbers of PV^+^ cells in the vehicle + static, MK801 + static, vehicle + run and MK801 + run groups were 55.11 ± 6.48,36.29 ± 4.40, 68.68 ± 5.7 and 50.69 ± 4.84 cells/mm^2^, respectively. One-way ANOVA, *F*_3_,_16_ = 30.39, *P* < 0.001; *post hoc* test: **P < 0.01, ***P < 0.001, ****P < 0.0001. **(D)** Quantitative analysis of the GFP-positive cell number in the DG. Data are expressed as mean ± SEM, and there were five mice in each group. Five sections in each animal were picked and counted. The numbers of GFP^+^ cells in the vehicle + static, MK801 + static, vehicle + run and MK801 + run groups were 43.01 ± 5.21,23.30 ± 4.12, 54.13 ± 4.13 and 34.15 ± 4.45 cells/mm^2^, respectively. One-way ANOVA, *F*_3_,_16_ = 42.41, *P* < 0.001; *post hoc* test: *P < 0.05; **P < 0.01, ***P < 0.001, ****P < 0.0001. **(E)** Representative images of the total branching dendrites in one GFP-positive cell in the DG. Scale bar, 200 μm. **(F)** Quantitative analysis of the total number dendritic branches in a GFP-positive cell. Data are expressed as mean ± SEM, and there were three mice in each group. Five sections in each animal were picked. Three cells in the DG from each section were analyzed and counted. The numbers of the total branching dendrites in one GFP-positive cell in the vehicle + static, MK801 + static, vehicle + run and MK801 + run groups were 7.43 ± 0.89, 2.6 ± 0.22, 9.34 ± 1.05 and 4.00 ± 0.40 cells/mm^2^, respectively. One-way ANOVA, *F*_3_,_16_ = 90.76, *P* < 0.001; *post hoc* test: *P < 0.05, **P < 0.01, ****P < 0.0001.)

To investigate whether PV interneurons regulate the development of adult newborn neurons, we labeled the latter with a retrovirus expressing GFP (shRNA-EF1a(s)-EGFP; Obio Technology, China) (Ge et al., 2006; Ma et al., 2009) by stereotaxic microinjection to DG 3 days before adaptive running (Figure 2A). GFP completely filled the soma, dendrites, and frequently the axons of adult-born granular cells (Figure 2B). Schizophrenia-like model ones (MK801 + static) decreased PV-positive cell numbers, accompanied by a drop in GFP-positive cells when compared to the control ones (vehicle + static). Running increased PV-positive and GFP-positive cell numbers, compared the vehicle + run group to the vehicle + static group, in line with previous studies (Arida et al., 2004, 2007). In schizophrenia-like model mice, running also significantly increased GFP-positive neurons and PV-positive neurons, almost back to normal numbers (Figures 2C,D). These findings implied that reduced neurogenesis in the hippocampus caused by MK801 injection might due to the reduced number of PV-positive interneuron, and treadmill running treatment restored adult neurogenesis by increasing the number of PV-positive interneuron.

Dendritic morphology is regarded as one important indicator of neuronal maturation (Livneh and Mizrahi, 2011; van der Velden et al., 2012). To further elucidate the effect of treadmill running on the maturation of newborn neurons, the total dendritic branches of GFP-positive neurons were analyzed by sholl analysis (Sholl, 1953; Figure 2E). Treadmill running significantly increased the number of total dendritic branches, and a significant decrease was observed in the DG after MK801 injection; however, treadmill running partially reversed the effect of MK801 injection on total dendritic branches of GFP-positive neurons in the DG (Figure 2F).

### Treadmill Running Ameliorates MK801-Induced Behavioral Changes

To investigate whether schizophrenia-like model mice could be reversed by treadmill running, we performed a series of behavior tests.

**FIGURE 3:**
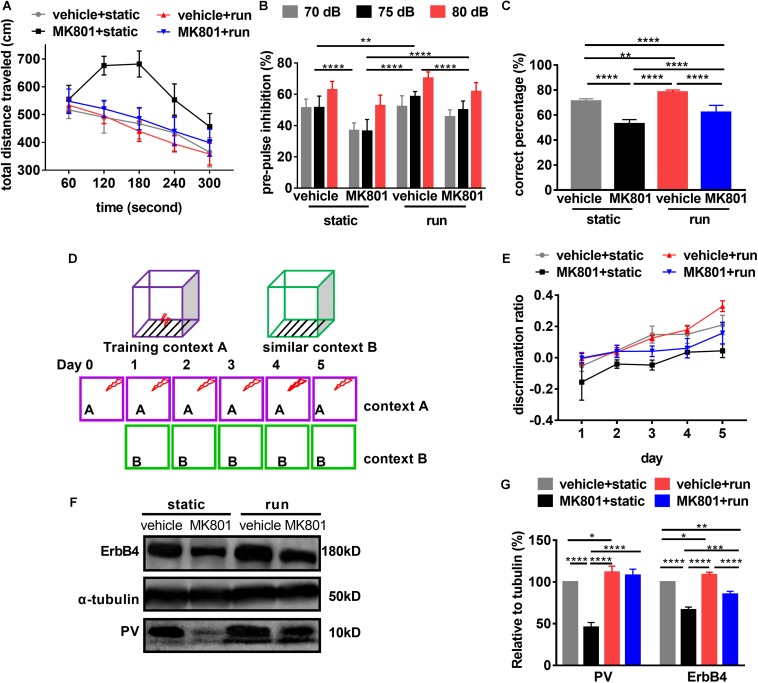
Schizophrenia-related behavioral phenotypes are reversed by running. **(A)** Hyperlocomotion induced by MK801 is inhibited by running. Data are expressed as mean ± SEM, and 3 were 10 mice in vehicle + static group, 10 mice in MK801 + static, 10 mice in vehicle + run group, and 14 mice in MK801 + run group, respectively. One-way ANOVA, *F*_3_,_16_ = 30.39, *P* < 0.001; *post hoc* test: vehicle + static vs. MK801 + static: *P* < 0.0001, vehicle + static vs. MK801 + run: *P* < 0.05, MK801 + static vs. vehicle + run: *P* < 0.0001, MK801 + static vs. MK801 + run: *P* < 0.0001, vehicle + run vs. MK801 + run: *P* < 0.001. **(B)** Treadmill running attenuates the prepulse inhibition deficit induced by MK801. Data are expressed as mean ± SEM, and 3 were 10 mice in vehicle + static group, 10 mice in MK801 + static, 10 mice in vehicle + run group, and 14 mice in MK801 + run group, respectively. Two-way ANOVA, *F*_3_,_117_ = 55.43, *P* < 0.001; *post hoc* test: **P < 0.01, ****P < 0.0001. **(C)** The working memory deficit induced by MK801 is attenuated by running. Data are expressed as mean ± SEM, and there were 10 mice in vehicle + static group, 10 mice in MK801 + static, 10 mice in vehicle + run group, and 14 mice in MK801 + run group, respectively. One-way ANOVA, *F*_3_,_37_ = 75.61, *P* < 0.001; *post hoc* test: **P < 0.01, ****P < 0.0001. **(D)** Mice were tested in a contextual fear discrimination learning paradigm. Briefly, in two similar contexts, foot shook was only present in context A. After several trials training in context A and B, mice could discriminate context A but not B as the cue of foot shook tested by freezing. **(E)** Pattern separation is improved by running. Data are expressed as mean ± SEM, and there were 10 mice in vehicle + static group, 10 mice in MK801 + static, 10 mice in vehicle + run group, and 14 mice in MK801 + run group, respectively. Two-way ANOVA, *F*_3_,_205_ = 117.3, *P* < 0.001; *post hoc* test: vehicle + static vs. MK801 + static: *P* < 0.0001, vehicle + static vs. vehicle + run: *P* < 0.01, vehicle + static vs. MK801 + run: *P* < 0.05, MK801 + static vs. vehicle + run: *P* < 0.0001, MK801 + static vs. MK801 + run: *P* < 0.0001, vehicle + run vs. MK801 + run: *P* < 0.001. **(F)** Western blotting for PV and ErbB4 in the hippocampus. a-Tubulin served as loading control. **(G)** Quantitative analysis of the Expression of PV and ErbB4 in the hippocampus. Data are expressed as mean ± SEM, and there were three mice in each group. Two-way ANOVA, *F*_3_,_16_ = 155.7, *P* < 0.001; *post hoc* test: *P < 0.05; **P < 0.01, ***P < 0.001, ****P < 0.0001.

First, we evaluated hyperactivity using OFT. *Post hoc* analysis revealed that the total distance traveled were not changed in the mice subjected to treadmill running (vehicle + run) compared to the control mice (vehicle + static). This is in line with the previous research (Zheng et al., 2019). Schizophrenia-like model mice (MK801 + static) showed hyperlocomotion compared to the control ones (vehicle + static). Treadmill running (MK801 + run) decreased the distances traveled in the arena almost back to normal (vehicle + static) (Figure 3A), providing further evidence that the hyperlocomotion phenotype of MK801 induced schizophrenia-like model mice can be rescued by treadmill running.

Second, we evaluated the effect of treadmill running on sensory gating using PPI. We found that PPI significantly increased with prepulse intensity in all groups, indicating that prepulse intensity can be distinguished by animals. *Post hoc* analysis revealed that PPI in the schizophrenia-like model mice (MK801 + static) was significantly lower than those control ones (vehicle + static). Treadmill running (MK801 + run) increased PPI when compared to the model mice (MK801 + static), but significantly lower than the control ones (vehicle + static) (Figure 3B). These observations demonstrate that running can rescue the MK801-induced sensory gating phenotype.

Third, we evaluated the effect of treadmill running on working memory using the T-maze test. The schizophrenia-like model mice (MK801 + static) performed significantly fewer correct entries when compared to those control ones (vehicle + static), indicating that MK801 administration impaired working memory. Treadmill running (vehicle + run) significantly increased the times of correct choices when compared to the control ones (vehicle + static). In the same way, treadmill running attenuated the working memory deficits induced by MK801 administration, but failed to restore performance to control levels (Figure 3C), indicating that running can only partially reverse working memory deficits.

Fourth, we examined the effect of treadmill running on pattern separation using contextual fear conditioning. It was shown that repeated MK801 administration impairs the ability of mice to discriminate similar environments (Mandillo et al., 2003). Since running improved the survival of newborn neurons after MK801 administration, we tested whether pattern separation deficits in the schizophrenia model could be improved by running. Thus, we performed a contextual fear discrimination learning paradigm, as summarized in Figure 3D (Sahay et al., 2011). All groups in training context A (with foot shock after 24 hr) showed elevated and indistinguishable levels of freezing, suggesting that all mice acquired and retained contextual fear equally. Negligible levels of freezing were observed in similar context B, which included no foot shock (data not shown). All mice learned to differentiate between context A and context B, resulting in a significant interaction between the freezing behaviors in the two contexts over time (Figure 3E). Running (vehicle + run) significantly increased the discrimination ratio between the two contexts when compared to those of control ones (vehicle + static), which is in line with the previous study (Creer et al., 2010). This discrimination ratio was significantly reduced by MK801 injection (MK801 + static). In the same way, this effect was improved by treadmill running (MK801 + run), while significantly lower than the control ones (vehicle + static) (Figure 3E). Hence, running improved pattern separation not only in normal mice but also in the schizophrenia-like model would provide additional evidence for a role of neurogenesis.

Previously, it has been reported that ErbB4 is a schizophrenia susceptibility gene. It is expressed in many neuronal populations (Stefansson et al., 2002; Yang et al., 2003; Mei and Xiong, 2008), mainly in PV-positive interneurons (Fazzari et al., 2010). ErbB4 is critical for maintaining PV neuron activity (Chen et al., 2010; Wen et al., 2010). Western blotting confirmed that both ErbB4 and PV expression was significantly down-regulated in the hippocampus after MK801 injection. Conversely, running significantly upregulated ErbB4 protein expression (Figures 3F,G). These results suggest ErbB4 and PV might be critical for the therapeutic effect of running to schizophrenia-related phenotypes.

### PV Interneuron Ablation in the DG Leads to Reduction in Adult Hippocampal Neurogenesis

**FIGURE 4:**
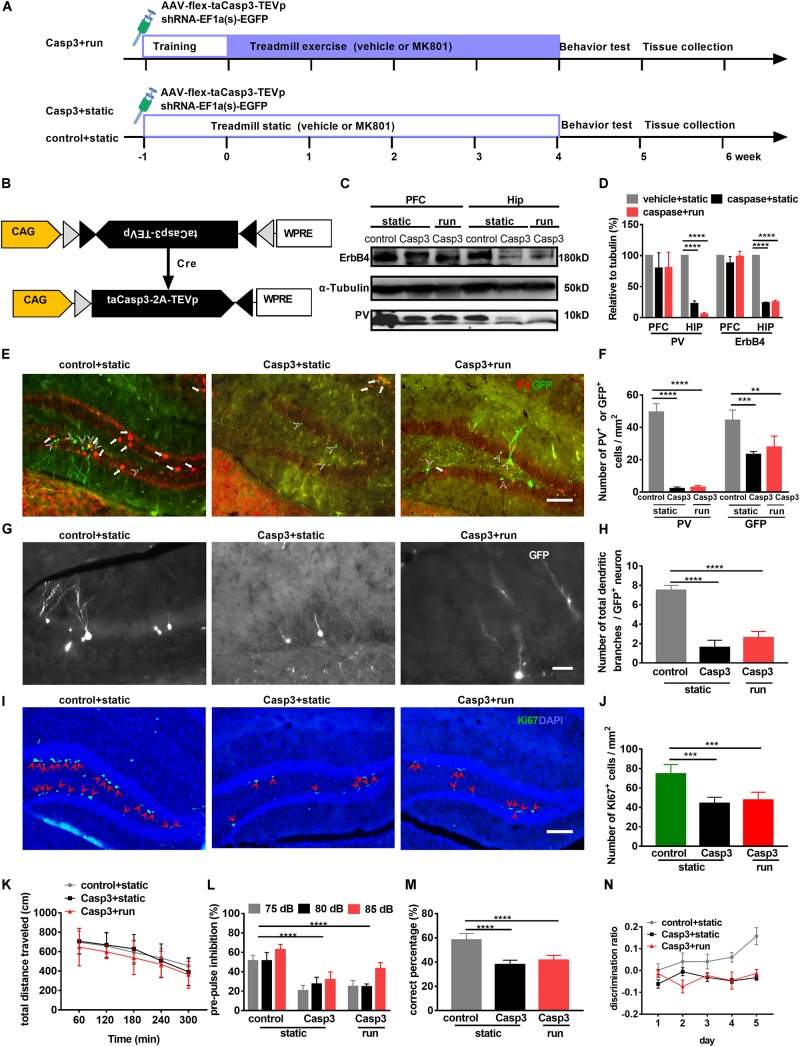
Treadmill running cannot improve adult neurogenesis and behavioral deficits induced by PV neuron ablation in the dentate gyrus (DG). **(A)** Experimental design for PV-Cre mice. Animals were stereotaxically injected with RV-U6-GFP to label newborn neurons (RV-GFP; green) and with AAV-flex-taCasp3 to ablate PV neurons. **(B)** Schematic illustration of AAV-flex-taCasp3-TEVp injection in PV-Cre mice to ablate PV neurons. **(C)** Representative western blots of PV and ErbB4 in (the hippocampus (Hip) and prefrontal cortex (PFC). a-Tubulin served as loading control. **(D)** Quantitative analysis of the Expression of PV and ErbB4 in the hippocampus and PFC. Data are expressed as mean ± SEM, and there were three mice in each group. Two-way ANOVA, *F*_2_,_24_ = 72.76, *P* < 0.001; *post hoc* test: ****P < 0.0001. **(E)** DREADD expression in PV neurons in the DG was identified by immunofluorescence staining for PV (red). Arrowheads indicate GFP-positive cells. Arrows indicate PV-positive interneurons. Scale bar, 100 μm. **(F)** Quantitative analysis of PV-positive interneurons and GFP-positive cells in the DG. Data are expressed as mean ± SEM, and there were five mice in each group. The numbers of PV + cells in the control + static, Casp3 + static and Casp3 + run groups were 49.46 ± 5.17, 2.15 ± 1.04 and 3.00 ± 0.98, respectively. The numbers of GFP + cells in the control + static, Casp3 + static and Casp3 + run groups were 44.35 ± 6.33, 23.29 ± 1.79 and 27.78 ± 6.85 cells/mm^2^, respectively. Two-way ANOVA, *F*_2_,_24_ = 182.2, *P* < 0.001; *post hoc* test: **P < 0.01, ***P < 0.001,****P < 0.0001. **(G)** Representative photomicrographs showing GFP-positive branching dendrites (white) in the DG. Scale bar, 200 μm. **(H)** Quantitative analysis of the total dendrite branch in a GFP-positive cell in the DG. Data are expressed as mean ± SEM, and there were three mice in each group, five sections in each animal were picked. Three cells in the DG from each section were analyzed and counted. The numbers of the total dendrite branch in a GFP-positive cell in the control + static, Casp3 + static and Casp3 + run groups were 7.50 ± 0.50, 1.60 ± 0.75 and 2.60 ± 0.64 branches/cell, respectively, Two-way ANOVA, *F*_2_,_12_ = 122.5, *P* < 0.001; *post hoc* test: ****P < 0.0001. **(I)** Representative photomicrographs showing Ki67- and DAPI-positive cells in the DG. Arrowheads (in red) indicated co-labeled cells (Ki67^+^DAPI^+^ cells) in the DG. Scale bar, 100 μm. **(J)** Running cannot reverse proliferating cell reduction, as marked by Ki67 expression, induced by PV neuron ablation in the DG. Data are expressed as mean ± SEM, and there were five mice in each group. The numbers of Ki67^+^ cell in the control + static, Casp3 + static and Casp3 + run groups were 7.50 ± 0.50, 1.60 ± 0.75 and 2.60 ± 0.64 cells/mm^2^, respectively, Two-way ANOVA, *F*_2_,_12_ = 21.06, *P* = 0.0001; *post hoc* test: ***P < 0.0001. **(K)** PV neuron ablation does not affect the locomotor activity in OFT. Data are expressed as mean ± SEM, and there were eight mice in each group. Two-way ANOVA, *F*_2_,_85_ = 2.305, *P* = 0.1060; *post hoc* test: There were no significant difference. **(L)** Deficits in prepulse inhibition induced by PV neuron ablation cannot be improved by running. Data are expressed as mean ± SEM, and there were eight mice in each group. Two-way ANOVA, *F*_2_,_51_ = 101, *P* < 0.0001; *post hoc* test: ****P < 0.0001. **(M)** Deficits in spatial working memory induced by PV neuron ablation (Casp3 + static group) cannot be improved by running (Casp3 + run group). Data are expressed as mean ± SEM, and there were eight mice in each group. One-way ANOVA, *F*_2_,_15_ = 12.23, *P* = 0.0007; *post hoc* test: ****P < 0.0001. **(N)** Pattern separation deficits induced by PV neuron ablation cannot be improved by running. Data are expressed as mean ± SEM, and there were eight mice in each group. Two-way ANOVA, *F*_2_,_115_ = 180.2, *P* < 0.0001; *post hoc* test: control + static vs. Casp3 + static*: P* < 0.0001, control + static vs. Casp3 + run*: P* < 0.0001.)

Recent studies have shown that PV-positive interneurons form immature synaptic inputs onto proliferating newborn progenitors, thus promoting their survival and maturation in the adult DG (Song et al., 2013). However, long-term consequences of altered hippocampal neurogenesis resulting from disrupted PV activity remain unknown. To further evaluate the relationship between PV and newborn cells, a Cre-dependent AAV expressing the pro-apoptotic protease Casp-3 (AAV-flex-taCasp3-TEVp, Taitool Bioscience, China; Figures 4A,B) (Yang et al., 2013; Zhang et al., 2016) and a retrovirus expressing GFP (RV-U6-GFP) were microinjected simultaneously into the hilus of adult PV-Cre mice 3 days before adaptive run-training to ablate PV neurons and label newborn cells, respectively (Figures 4A,B). Animals were randomly divided into three groups: (1) mice injected with a control virus (AAV-DIO-mCherry) and static on the treadmill (control + static group), (2) animal injected with AAV-flex-taCasp3-TEVp and static on the treadmill (Casp3 + static group), and (3) animals injected with AAV-flex-taCasp3-TEVp and subjected to treadmill running (Casp3 + run group).

Western blot analysis (Figures 4C,D) confirmed that ErbB4 and PV levels were significantly decreased in the hippocampus of *PV-taCasp3* mice, while almost no PV interneuron in the DG was observed even 42 days after virus injection (Figures 4E,F). Moreover, a significant decrease in PV-positive and GFP-positive cell numbers in the DG was noted in PV-taCasp3 mice (Casp3 + static and Casp3 + run) compared to those of control ones (control + static) (Figures 4E,F). Interestingly, no difference was observed in GFP-positive cell number between the Casp3 + static and Casp3 + run groups (Figure 4F), suggesting that caspase-induced neuron ablation was efficient and specific to PV interneurons, and this ablation leads to reduction of newborn cells.

To further elucidate the effect of PV interneurons ablation on the maturation of newborn cells, the total dendritic trees of GFP-positive neurons were observed. GFP-positive dendritic branches were sharply reduced (Figures 4G,H). The number of GFP-positive dendritic branches was significantly lower in PV-taCasp3 mice (Casp3 + static and Casp3 + run) when compared to those control ones (control + static); however, there were no difference between the static and running group of PV-taCasp3 mice (Figure 4I), indicating that PV interneurons ablation imposes restrictions on adult hippocampal neurogenesis. The number of proliferating cells marked by Ki67 was also significantly reduced after PV ablation in the two groups with PV-taCasp3 mice (Figures 4I,J), suggesting that PV ablation leads to impaired proliferation. These results further indicate that PV-positive interneurons involved in neurogenesis induced by running.

### PV Interneuron Ablation in the DG Exacerbates Abnormal PPI, Cognitive Impairment, and Discrimination Deficits

To test whether PV interneurons in the DG are required for the positive effects observed in schizophrenia-like model mice by treadmill running, we compared the PV-taCasp3 mice with their littermate wild-type control mice on a series of experiments, including the OFT, PPI, and T-maze. Animals were randomly divided into three groups: (1) control mice with static (control + static), (2) PV-taCasp3 mice with static (Casp3 + static), and (3) PV-taCasp3 mice with run (Casp3 + run).

In the OFT, the exploratory behavior was similar for all three groups of mice, indicating that PV interneurons ablation in DG does not result from lower or higher motor abilities (Figure 4K). However, PV-positive interneuron ablation in the DG (Casp3 + static) significantly decreased PPI and working memory when compared to those control ones (control + static), and these effects could not be reversed by running (Casp3 + run) (Figures 4L,M). Furthermore, when PV interneurons were ablated, the pattern separation deficits persisted, the discrimination ratio of PV-taCasp3 mice with static (Casp3 + static) being significantly decreased when compared to those control ones (control + static) and are not significantly different from mice subjected to treadmill running (Casp3 + run) (Figure 4N). These data indicate that PV interneurons are indispensable for reversing cognitive function deficits.

### Activation of PV Neurons of the DG Is Sufficient to Improve Schizophrenia-Related Behaviors and Neurogenesis

**FIGURE 5:**
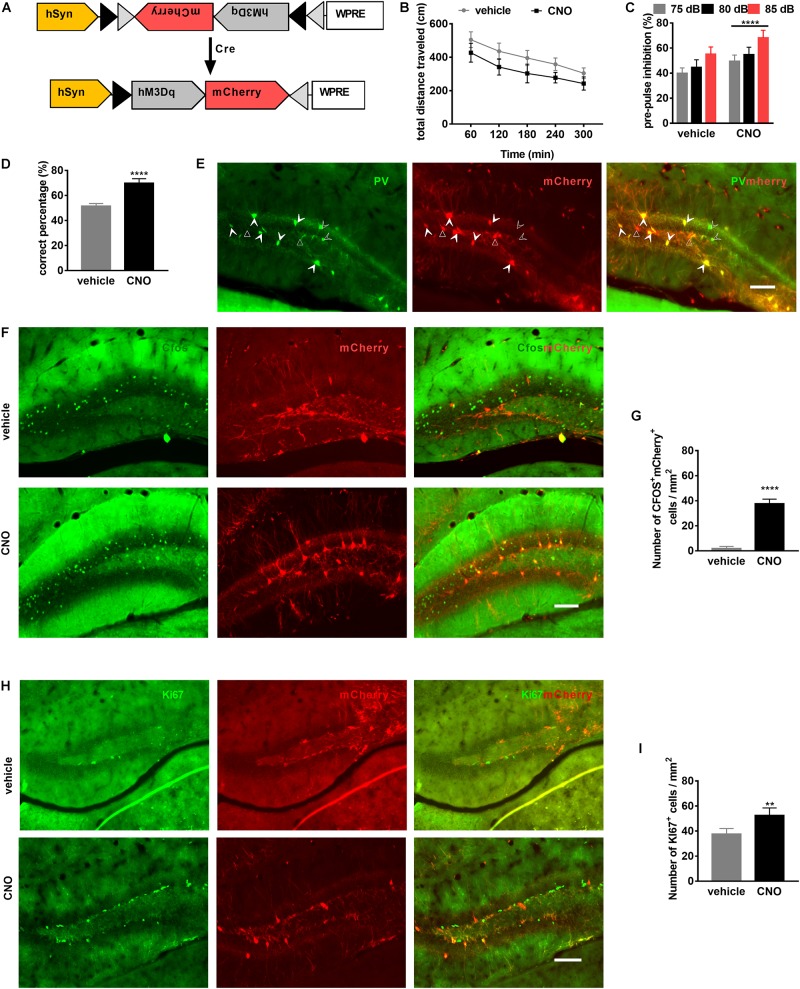
Activation of PV neurons in the dentate gyrus (DG) is sufficient to attenuate schizophrenia-like behavioral phenotypes. **(A)** Schematic illustration of AAV-hSyn-DIO-hM3DGq-mCherry injection in PV-Cre mice to express the DREADD receptor. **(B)** Locomotor activity of PV-DG^hM3Dq^ mice in the OFT is improved by clozapine-N-oxide (CNO) treatment. Data are expressed as mean ± SEM, and there were eight mice in each group. Two-way ANOVA, *F*_2_,_110_ = 98.58, *P* < 0.0001; *post hoc* test: vehicle vs. CNO: *P* < 0.0001. **(C)** Prepulse inhibition of PV-DG^hM3Dq^ mice is improved by CNO treatment. Data are expressed as mean ± SEM, and there were eight mice in each group. Two-way ANOVA, *F*_1_,_42_ = 47.45, *P* < 0.0001; *post hoc* test: ****P < 0.0001. **(D)** Spatial working memory of PV-DG^hM3Dq^ mice (is improved by CNO treatment. Data are expressed as mean ± SEM, and there were eight mice in each group. Unpaired *T*-test: ****P < 0.001. **(E)** Representative photomicrographs showing PV-positive interneurons (in green) and mCherry-positive cells (in red) in the DG. Unfilled triangles indicate mCherry-positive cells in the DG. Unfilled arrowheads indicate PV-positive interneurons. Filled Arrowheads indicate PV^+^mCherry^+^ co-labeled interneurons. Scale bar, 100 μm. **(F)** Representative photomicrographs showing mCherry^+^ cells (in red), c-Fos^+^ cells (in green), and their overlay (in yellow) in the DG. Scale bar, 100 μm. **(G)** CNO activates PV-hM3Dq^+^ interneurons in the DG. Data are expressed as mean ± SEM, and there were five mice in each group. Unpaired *T*-test: ****P < 0.0001. **(H)** Representative photomicrographs showing mCherry^+^ cells (in red) and Ki67-positive cells (in green) in the DG. Scale bar, 100 μm. **(I)** PV-hM3Dq^+^ interneurons are activated by CNO-induced neurogenesis. Data are expressed as mean ± SEM, and there were five mice in each group. Unpaired *T*-test: **P < 0.01.)

To examine whether PV-interneuron activation in the DG improves schizophrenia-related behaviors, DREADD (Designer Receptors Exclusively Activated by Designer Drugs) technology was used (Alexander et al., 2009). Briefly, the Cre-dependent AAV-DIO-hM3DGq-mCherry (Figure 5A) was bilaterally injected (0.4 μL per hemisphere, titer: 1 × 10^12^, GeneChem technologies, China) into the DG of PV-Cre mice (PV-DG^hM3Dq^ mice) (Figure 5A). Animals were randomly divided into two groups: (1) mice injected with vehicle (vehicle group) and 2) those injected with clozapine-N-oxide (CNO; CNO group).

To investigate whether PV-interneuron activation could improve behavior, we performed a series of behavior tests. CNO treatment decreased the total distance traveled in the OFT (Figure 5B), in the same way, PPI and spatial working memory were also improved by CNO treatment (Figures 5C,D).

Cre dependent expression of mCherry was confirmed by immunofluorescence staining for PV. The PV-positive interneurons in the DG were co-localized with mCherry-positive cells (Figure 5E). To observe PV-positive interneurons activated by CNO, we performed immunofluorescence staining for c-Fos, the immediate early gene, a marker for neuronal activation in the brain (Hoffman et al., 1993). After CNO administration, mCherry and c-Fos co-localized expression confirmed activation of PV interneurons (Figure 5F). *Post hoc* analysis confirmed the number of mCherry^+^c-Fos^+^ cells were significantly increased by CNO compared to vehicle (Figure 5G). To evaluate whether PV-interneuron activation can improve neurogenesis, proliferating cells, marked by Ki67, in the DG were also observed (Figure 5H). *Post hoc* analysis confirmed the number of Ki67-positive cells was also significantly increased by CNO activation (Figure 5I). These results suggested that PV-interneuron activation in the DG might represent an efficient strategy to improve schizophrenia-related behaviors and neurogenesis.

### Treadmill Running Ameliorates Behavioral Changes of PV-ErbB4^–/–^ Mice

**FIGURE 6:**
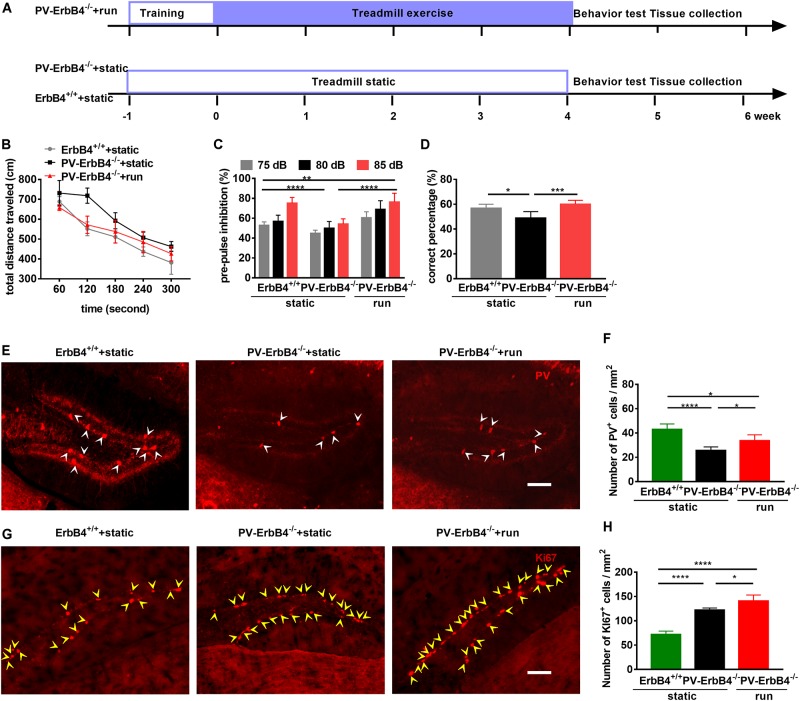
Treadmill running improves schizophrenia-related behavioral phenotypes and increases adult neurogenesis in the dentate gyrus (DG) of PV-ErbB4^–/–^ mice. **(A)** Schematic experimental design for PV-ErbB4^–/–^ mouse. **(B)** Hyper-locomotion of PV-ErbB4^–/–^ mice is inhibited by running. Data are expressed as mean ± SEM, and there were six mice in ErbB4^+/+^ + static group, nine mice in PV-ErbB4^–/–^ + static group and six mice in PV-ErbB4^–/–^ + run group. Two-way ANOVA, *F*_2_,_90_ = 42.79, *P* < 0.0001; *post hoc* test: ErbB4^+/+^ + static group vs. PV-ErbB4^–/–^ + static: *P* < 0.0001, PV-ErbB4^–/–^ + static group vs. PV-ErbB4^–/–^ + run: *P* < 0.0001. **(C)** Prepulse inhibition deficits in PV-ErbB4^–/–^ mice are attenuated by treadmill running. Data are expressed as mean ± SEM, and there were six mice in ErbB4^+/+^ + static group, nine mice in PV-ErbB4^–/–^ + static group and six mice in PV-ErbB4^–/–^ + run group. Two-way ANOVA, *F*_2_,_90_ = 42.79, *P* < 0.0001; *post hoc* test: **P < 0.01, ****P < 0.0001. **(D)** Working memory deficits in PV-ErbB4^–/–^ mice are attenuated by running. Data are expressed as mean ± SEM, and there were six mice in ErbB4^+/+^ + static group, nine mice in PV-ErbB4^–/–^ + static group and six mice in PV-ErbB4^–/–^ + run group. Two-way ANOVA, *F*_2_,_51_ = 47.38, *P* < 0.0001; *post hoc* test: *P < 0.05, ***P < 0.001. **(E)** Representative photomicrographs showing PV-positive interneurons in the DG. Arrowheads (in white) indicate PV-positive cells in the DG. Scale bar, 100 μm. **(F)** Quantitative analysis of PV-positive cells in the DG. Data are expressed as mean ± SEM, and there were five mice in each group. One-way ANOVA, *F*_2_,_12_ = 21.78, *P* = 0.0001; *post hoc* test: ***P < 0.001, ****P < 0.0001. **(G)** Representative photomicrographs showing Ki67-positive cells in the DG. Arrowheads (in yellow) indicate Ki67-positive cells in the DG. Scale bar, 100 μm. **(H)** Quantitative analysis of Ki67-positive cells in the DG. Data are expressed as mean ± SEM, and there were five mice in each group. One-way ANOVA, *F*_2_,_12_ = 89.86, *P* < 0.0001; *post hoc* test: *P < 0.05, ****P < 0.0001.

To investigate whether the ErbB4 receptor is involved in antipsychotic effect of treadmill running, we performed schizophrenia-related behavior tests in PV-ErbB4^–/–^ mice (Figure 6A). PV-ErbB4^–/–^ mice has been shown to increase locomotor activity, disrupt sensorimotor gating (PPI) and cognitive function, and impair T-maze spatial working memory (Wen et al., 2010). Treadmill running (PV-ErbB4^–/–^ + run) decreased the distance traveled in the OFT (Figure 6B), increased PPI (Figure 6C), attenuated the working memory deficits, and even restored T-maze performance back to control levels when compared to those corresponding model mice (PV-ErbB4^–/–^ + static) (Figure 6D). This provides further evidence that these phenotypes, partially comparable to those in schizophrenic patients, can be rescued by treadmill running in mice.

### Treadmill Running Increases Neurogenesis and PV-Positive Interneurons in the DG of PV-ErbB4^–/–^ Mice

To determine the effect of running on PV-positive interneurons in PV-ErbB4^–/–^ mice, we used immunofluorescence staining for PV interneurons. The membrane and cytoplasm of PV interneurons in the DG of control mice were strongly immunoreactive by immunostaining, having a fusiform or elliptical shape (Figure 6E). *Post hoc* analysis revealed that ErbB4 ablation significantly decreased PV-positive cells number (Figure 6F), which is in line with previous reports (Chen et al., 2010; Tian et al., 2017; Zhang et al., 2018). PV-ErbB4^–/–^ mice subjected to treadmill running (PV-ErbB4^–/–^ + run) increased the number of PV-positive interneurons when compared to those corresponding model mice (PV-ErbB4^–/–^ + static) (Figure 6F).

ErbB4 ablation in PV interneurons sharply increase the proliferation of NSCs in the hippocampus (Figures 6G,H), in line with previous reports using BrdU labeling (Zhang et al., 2018). Moreover, PV-ErbB4^–/–^ mice subjected to treadmill running significantly increased the number of Ki67-positive cells in the DG (Figure 6H). Intriguingly, treadmill running also increased the number of PV-positive neurons in the DG (Figures 6F,H), suggesting the involvement of PV interneuron and neurogenesis in the antipsychotic effect of treadmill running.

## Discussion

Our study confirms that PV neurons in the DG are critical for the action of treadmill running in reversing schizophrenia-like phenotypes by promoting adult neurogenesis. First, deficits in adult neurogenesis in the MK801-induced schizophrenia model were partially reversed by treadmill running both through increased NSC proliferation and generation of new neurons in the DG. Interestingly, PV-neuron number and their activity in the DG were also decreased in the schizophrenia model and were fully restored by treadmill running. In parallel, schizophrenia-related behavioral phenotypes induced by MK801 were ameliorated by treadmill running. Finally, PV-neuron ablation in the DG significantly decreased adult neurogenesis and induced cognitive impairments, which could not be reversed by treadmill running.

Aerobic exercise has been shown to improve both “positive symptoms” of schizophrenia, including delusions and hallucinations, and “negative symptoms,” including cognitive inability and emotion blunt (Beebe et al., 2005; Duraiswamy et al., 2007; Vancampfort et al., 2011, 2017; Scheewe et al., 2013; Firth et al., 2017; Kurebayashi and Otaki, 2017). Since negative symptoms are almost not amenable with antipsychotic drug treatment (Fusar-Poli et al., 2015; Leucht et al., 2017), non-pharmacological interventions, including aerobic exercise and enriched environment, are needed to achieve better outcomes. Although the mechanism by which exercise improves schizophrenia symptoms is still largely unknown, it is well accepted that moderate exercise increases adult neurogenesis, and that aberrant neurogenesis contributes to schizophrenia symptoms. In this study, MK801 induced aberrant neurogenesis in the DG and schizophrenia-related behavioral phenotypes that were almost fully reversed by moderate treadmill running (Figure 1), suggesting that NSCs in the SGZ are critical mediators of the effect of exercise in rescuing schizophrenia symptoms.

Exercise was shown to increase adult neurogenesis through neurotrophins, including BDNF (Vaynman et al., 2004) and insulin-like growth factor 1 (Trejo et al., 2001). BDNF promotes adult neurogenesis through GABAergic neurons, including PV neurons (Waterhouse et al., 2012). It is well accepted that hypofunction of PV neurons causes schizophrenia-like symptoms (Nakazawa et al., 2012), and that anti-psychotic medications, including fluoxetine and clozapine, have protective effects on PV neurons (Filipovic et al., 2018). In the present study, exercise preserved the number and activity of PV neurons at normal levels after MK801 treatment, suggesting that PV neurons play a critical role in exercise-induced adult neurogenesis, reversing the schizophrenia-related behavioral phenotypes.

Parvalbumin-positive interneurons associated in close proximity with the adult-born GCs (Gupta et al., 2019), form immature synaptic inputs onto proliferating newborn progeny and promote newborn neuronal progeny survival and development in the adult DG (Song et al., 2013), while suppressed PV-interneuron activity decreases the survival and maturation of newborn neurons (Song et al., 2013). In the present study, PV neurons were also spotted very close to NSCs. However, the adult neurogenesis deficit induced by their elimination in the DG could not be rescued by treadmill running, indicating that PV is required for exercise to promote adult neurogenesis. Newborn neurons mature and form functional synapses with their efferent targets from CA2 and CA3 pyramidal neurons, and receive synaptic information from the perforant pathway and from inhibitory interneurons (Zhao et al., 2006; Toni et al., 2008; Llorens-Martin et al., 2015; Alvarez et al., 2016). Therefore, these newborn neurons are pivotal for the normal function of the DG, and any impairment in their survival and maturation will induce various schizophrenia-related cognitive deficits.

Exercise promotes adult neurogenesis in the SGZ (van Praag et al., 1999; Kronenberg et al., 2003), and several studies have investigated the potential implications of decreased adult neurogenesis in this region in patients with schizophrenia (Pereira et al., 2007; Allen et al., 2016). Intriguingly, ablating PV neurons only in the DG is enough to elicit schizophrenia-related cognitive behavioral phenotypes, suggesting that PV neurons and adult neurogenesis in the SGZ are required for the therapeutic effect of exercise on schizophrenia. Moreover, excitation of PV-positive interneurons in the cortex and hippocampus contributes to normal cognitive functions in mice (Yi et al., 2014). Our results show that activation of PV-positive neurons only in the DG using chemogenetic technology reduces hyperlocomotion and improves PPI, working memory, and neurogenesis. These results suggest that PV-interneuron activation in the DG represents a good strategy to rescue schizophrenia-like phenotypes. Although such activation was reported to inhibit quiescent NSCs (Song et al., 2012), we found that exercise increases PV interneurons number and adult neurogenesis, which might due to the different strategies employed to activate PV interneurons. So our findings suggest activation of PV interneurons in the DG might be a new strategy to reverse schizophrenia-like phenotypes.

## Data Availability Statement

All datasets generated for this study are included in the article/supplementary material.

## Ethics Statement

The animal study was reviewed and approved by the Animal Welfare Committee of Huazhong University of Science and Technology.

## Author Contributions

YL conceived the study and participated in the experiment design. YY performed the experiments, carried out the functional analysis, and drafted the manuscript. YS contributed to the experiment design and manuscript preparation.

## Conflict of Interest

The authors declare that the research was conducted in the absence of any commercial or financial relationships that could be construed as a potential conflict of interest.

## Footnotes

1 http://ghoshlab.org/software/
